# Synergistic Eradication of Drug-Resistant *Salmonella enteritidis* in Food Matrices Through an Ag-MOF Nanozyme with Multiple Enzyme-like Activities

**DOI:** 10.3390/foods15030479

**Published:** 2026-01-30

**Authors:** Baizhi Cen, Juge Liu, Mengyuan Tan, Bo Wang, Lu Gao, Zhenquan Yang, Genxi Zhang, Tao Zhang, Xuechao Xu

**Affiliations:** 1 School of Food Science and Engineering, Yangzhou University, Yangzhou 225127, China; 19825305779@163.com (B.C.); liujuge@yzu.edu.cn (J.L.); tanmy1119@163.com (M.T.); wb@yzu.edu.cn (B.W.); gaolu@yzu.edu.cn (L.G.); yangzq5730@163.com (Z.Y.); 2 College of Animal Science and Technology, Yangzhou University, Yangzhou 225127, China; gxzhang@yzu.edu.cn

**Keywords:** Ag-MOF nanozyme, multiple enzyme-like activities, synergistic eradication, drug-resistant *Salmonella enteritidis*, food matrices

## Abstract

In this study, a silver-based metal–organic framework (Ag-MOF) nanozyme was synthesized for the synergistic eradication of drug-resistant *Salmonella enteritidis* in food matrices. Ag-MOF exhibits multiple enzyme-like activities, namely oxidase (OXD)-, peroxidase (POD)-, and superoxide dismutase (SOD)-like activities. It demonstrated excellent antibacterial and antibiofilm activities against erythromycin- and chloramphenicol-resistant *S. enteritidis* strains (N29 and P23). Specifically, treatment with 20 mg mL^−1^ Ag-MOF resulted in nearly complete eradication of *S. enteritidis* in in vitro suspension assays, including 1 × 10^7^ CFU mL^−1^ N29 strain and 6 × 10^6^ CFU mL^−1^ P23 strain. Moreover, treatment with 1 mg mL^−1^ Ag-MOF led to 80~90% biofilm inhibition of *S. enteritidis*. Mechanistic investigations revealed that Ag-MOF effectively interacted with amino-rich structures on the bacterial surface (such as membrane proteins and peptidoglycan components), generated abundant reactive oxygen species (ROS), released Ag^+^ ions, and depleted intracellular glutathione, which collectively disrupted cell membrane integrity and induced severe leakage of intracellular proteins and nucleic acids. Importantly, Ag-MOF maintained high antibacterial efficacy in complex simulated food matrices (pork, milk, and egg shell). Overall, this study offers key insights into enzyme-mimicking antibacterial materials and a promising strategy to combat multidrug resistant foodborne pathogens.

## 1. Introduction

*Salmonella enteritidis* is a major foodborne pathogen that poses a serious global public health concern [1,2,3]. It is commonly linked to contaminated poultry, eggs, dairy products, and vegetables. Infection with *S. enteritidis* causes salmonellosis, a gastrointestinal disease manifesting as diarrhea, abdominal cramps, fever, nausea, and vomiting [4,5,6]. In vulnerable populations, including young children, the elderly, and immunocompromised individuals, the infection can cause more severe outcomes, with the pathogen potentially spreading beyond the gastrointestinal tract to induce systemic infections and life-threatening complications [7].

The emergence and rapid spread of drug-resistant *S. enteritidis* have exacerbated this public health burden. These resistant strains display multidrug resistance (MDR), greatly reducing the efficacy of conventional therapies [8]. Consequently, treatment becomes more challenging, often resulting in prolonged infection, therapeutic failure, increased hospitalization, and higher mortality rates [9]. Moreover, resistant *S. enteritidis* can circulate among humans, animals, and the environment, perpetuating its prevalence as a global zoonotic threat [10,11]. The indiscriminate use of antimicrobials in both clinical settings and animal farming has accelerated the evolution and dissemination of antimicrobial resistance in this pathogen [12]. Various antibacterial strategies, such as antibiotics, chemical disinfectants, physical treatments (e.g., heat or irradiation), and biological control agents, have been applied to combat *S. enteritidis*. However, their efficacy is often limited against resistant strains. Antibiotics, the cornerstone of salmonellosis therapy, frequently fail against MDR isolates [13]. Chemical disinfectants may leave toxic residues and exhibit reduced efficiency, while physical interventions, though effective, are energy-intensive and may compromise food quality [14]. Biological control approaches show certain potential but are often restricted by narrow antibacterial spectra and limited activity against diverse resistant strains [15,16].

Recently, nanotechnology-based antibacterial systems have gained growing attention as sustainable and efficient alternatives. In particular, nanozymes, engineered nanomaterials with intrinsic enzyme-like catalytic properties, have emerged as a powerful class of antimicrobial agents [17,18,19]. By combining the robustness and tunability of nanomaterials with the catalytic efficiency of natural enzymes, such as oxidase (OXD), peroxidase (POD), catalase (CAT), and superoxide dismutase (SOD), nanozymes can participate in redox reactions that generate reactive oxygen species (ROS) or decompose toxic intermediates [20,21,22]. Moreover, extensive research has demonstrated their potent antibacterial capabilities, even against MDR bacteria [23,24]. Unlike traditional antimicrobials that target specific biomolecules, nanozymes employ multiple antibacterial mechanisms, including the generation of ROS to damage bacterial membranes, proteins, and nucleic acids, disruption of metabolic pathways, and inhibition of biofilm formation [25,26,27]. This multi-pathway antibacterial mechanism makes it far more difficult for bacteria to develop resistance, positioning nanozymes as highly promising alternatives for combating MDR pathogens. Notably, silver-based metal–organic frameworks (Ag-MOF) have attracted significant attention due to their structural versatility, uniform distribution of active sites, and the synergistic advantages of MOF porosity and silver-mediated antibacterial activity [28].

In this study, we propose the design of an Ag-MOF nanozyme as a potential antibacterial platform for controlling drug-resistant *S. enteritidis* in food matrices. The Ag-MOF is designed to exhibit multiple enzyme-like activities, including OXD-, POD-, and SOD-like functions, which may collectively regulate ROS generation and redox processes. This enables not only the effective eradication of planktonic bacteria but also significant suppression of biofilm formation. The novelty lies in the construction of a multifunctional Ag-MOF nanozyme and the systematic investigation of its catalytic characteristics, antibacterial performance, and potential relevance in complex food-related systems. This study aims to provide mechanistic insights into nanozyme-based antibacterial strategies and to offer a conceptual framework for the development of alternative antimicrobial materials in food safety applications.

## 2. Materials and Methods

### 2.1. Materials and Reagents

Acetic acid (HAc), ethanol, sodium acetate (NaAc), triethylamine, methanol solution, N, N-dimethylformamide (DMF), and NaCl were sourced from Sinopharm Chemical Reagent Co., Ltd. (Shanghai, China). Silver nitrate (AgNO_3_), terephthalic acid (BDC), 3,3′,5,5′-tetramethylbenzidine (TMB), oleyl amine, oleic acid, oleyl alcohol, and dichloromethane were acquired from Aladdin Biochemical Technology Co., Ltd. (Shanghai, China). Agar powder medium, reduced glutathione (GSH), erythromycin, chloramphenicol, fluorescent dye propidium iodide (PI), and crystal violet were purchased from Soleibao Co., Ltd. (Beijing, China). Fluorescent dye SYBR Green, tryptone, and yeast extract were obtained from Thermo Fisher Scientific, Inc. (Shanghai, China). The SOD Assay Kit (NBT method) was procured from Beyotime Co., Ltd. (Shanghai, China). All reagents used in this study were of analytical grade and were employed without further purification. Deionized water was used throughout all experimental procedures.

### 2.2. Bacterial Culture

*S. enteritidis* strains N29 (CICC 21489) and P23 (CICC 21498) were incubated overnight at 37 °C in self-prepared Luria–Bertani (LB) broth [29].

### 2.3. Preparation of Ag-MOF

The Ag-MOF was synthesized via a slightly modified solvothermal method based on previously reported procedures [30]. Specifically, 1 mmol AgNO_3_, 2 mmol BDC, and 4 mmol triethylamine were dissolved in 50 mL methanol solution (50%). The mixture was vigorously stirred for 30 min and then allowed to stand undisturbed for 12 h to facilitate crystallization. The resulting precipitate was collected by centrifugation, washed repeatedly with methanol and deionized water, and dried under vacuum at room temperature for 24 h. According to inductively coupled plasma-optical emission spectrometry (ICP-OES, Thermo Fisher iCAP PRO) analysis, the silver content of the obtained Ag-MOF was determined to be 6.53 ± 0.15 wt%.

### 2.4. Characterization

Characterization of samples was conducted by using the following equipment. X-ray diffraction patterns (XRD) were performed on a D8 Advance polycrystalline X-ray diffractometer (Bruker, Karlsruhe, Germany). Transmission electron microscopy (TEM) images were obtained on a Tecnai 12 transmission electron microscopy (Philips, Eindhoven, The Netherlands). N_2_ adsorption–desorption experiments were performed by an automatic specific surface area tester (Jingweigaobo, Beijing, China). X-ray photoelectron spectroscopy (XPS) measurements were conducted on an AXIS ULTRA DLD X-ray photoelectron spectrometer (Shimadzu, Kyoto, Japan). Free radicals were ascertained by an electron paramagnetic resonance spectrometer (EPR, model A300-10/12, Bruker, Karlsruhe, Germany). Fluorescent imaging analysis was performed using a laser scanning confocal microscope (LSCM 880NLO, Carl Zeiss, Oberkochen, Germany). TMB chromogenic reaction was analyzed using a UV-Vis spectrophotometer (Evolution One, Thermo Scientific, Madison, WI, USA).

### 2.5. Multiple Enzyme-like Activities of Ag-MOF

The enzyme-mimicking activities of Ag-MOF were evaluated using the TMB chromogenic reaction and the xanthine–xanthine oxidase system [31]. To assess oxidase-like activity, 100 μL Ag-MOF suspension (1.5 mg mL^−1^) was mixed with 100 μL TMB solution (5 mmol L^−1^, dissolved in ethanol), followed by the addition of 2800 μL NaAc-HAc buffer (0.2 mol L^−1^, pH 4.0). After incubation at room temperature for 10 min, the reaction solution was immediately analyzed with a UV-Vis spectrophotometer. Peroxidase-like activity was determined by combining 100 μL Ag-MOF suspension (1.5 mg mL^−1^), 100 μL H_2_O_2_ solution (100 mmol L^−1^), and 100 μL TMB solution (5 mmol L^−1^, dissolved in ethanol), followed by the addition of 2700 μL NaAc-HAc buffer (0.2 mol L^−1^, pH 4.0). After 10 min of incubation at room temperature, the absorbance was recorded using a UV-Vis spectrophotometer. Superoxide dismutase-mimetic activity was evaluated using a commercial xanthine/xanthine oxidase detection kit (NBT) obtained from Beyotime Co., Ltd. (Shanghai, China). In accordance with the manufacturer’s instructions, 20 μL Ag-MOF suspension at various concentrations (1.0, 0.5, 0.25, 0.2, 0.1, 0.05, and 0 mg mL^−1^) was combined with the assay components, allowed to react for 30 min at room temperature, and subsequently measured using UV-Vis spectroscopy.

### 2.6. Eradication Activity of S. enteritidis

Eradication activity of *S. enteritidis* was determined according to the previously reported work [32].

The antibacterial activity of Ag-MOF against drug-resistant *S. enteritidis* was evaluated using a plate-counting assay. Briefly, 100 μL of Ag-MOF suspension (20 mg mL^−1^, prepared in physiological saline) or antibiotics (20 mg mL^−1^, prepared in physiological saline) and 100 μL of *S. enteritidis* suspension (N29 strain: 1 × 10^8^, 1 × 10^7^, 1 × 10^6^, 1 × 10^5^, 1 × 10^4^, 1 × 10^3^, and 1 × 10^2^ CFU mL^−1^ and P23 strain: 6 × 10^7^, 6 × 10^6^, 6 × 10^5^, 6 × 10^4^, 6 × 10^3^, 6 × 10^2^ and 6 × 10^1^ CFU mL^−1^) were added to 800 μL of physiological saline. The mixture was maintained at 37 °C for 2 h. Following this incubation period, 100 μL of mixture was uniformly plated onto homemade LB agar and subsequently incubated at 37 °C for 24 h until distinct colonies became visible.

The ability of Ag-MOF to inhibit *S. enteritidis* biofilm formation was assessed via crystal violet staining. A 96-well plate was used to combine 100 μL of bacterial suspension (10^8^ CFU mL^−1^) with 100 μL of physiological saline containing varying concentrations of Ag-MOF or antibiotics (1.0, 0.5, 0.25, 0.1, 0.05, and 0.01 mg mL^−1^). The mixture was then incubated at 37 °C for 54 h to facilitate biofilm growth. After that, each well was washed three times with deionized water and air-dried. Then, 200 μL methanol was added to fix the adherent bacteria for 15 min. Subsequently, 200 μL 0.05% crystal violet (*w*/*v*) staining solution was added, followed by washing with deionized water and air-drying. Subsequently, 200 μL glacial acetic acid (30%) was introduced to dissolve the crystal violet dye. Following a 10 min incubation period, the contents from each well were carefully transferred into a fresh 96-well plate. Biofilm biomass was quantified by measuring the optical density at 594 nm (OD_594_) using an Infinite F50microplate reader (TECAN, Männedorf, Switzerland). The inhibition rate was determined using the formula Inhibition rate (%) = (A_n_ − A_t_)/(A_n_ − A_b_) × 100%, where A_n_ indicates the average absorbance of the negative control group, A_t_ signifies the absorbance value of the treatment group, and A_b_ stands for the mean absorbance of the blank control group.

### 2.7. Eradication Mechanism of S. enteritidis

#### 2.7.1. Affinity Assessment Between Ag-MOF and Functional Groups

An amount of 2 mL of Ag-MOF suspension (1 mg mL^−1^) was added to 4 mL of dichloromethane, followed by the addition of 2 mL of individual reagents (oleyl amine, oleic acid, or oleyl alcohol). The resulting mixtures were sonicated for 10 min and incubated for 4 h.

#### 2.7.2. ROS Production Assessment

A 1 mL suspension of *S. enteritidis *(10^8^ CFU mL^−1^) was combined with 1 mL of Ag-MOF suspension (1 mg mL^−1^, prepared in physiological saline). Subsequently, 80 μL of 2′,7′-dichlorofluorescin diacetate (DCFH-DA, 10 μM) was introduced into the mixture. The sample was then incubated at 37 °C for 30 min under dark conditions, followed by immediate capture of fluorescence images using a fluorescence microscope.

#### 2.7.3. Assessment of GSH Depletion

Reduced GSH solution (0.2 mg mL^−1^) was mixed with Ag-MOF suspensions (0.25, 0.05, 0.1, 0.5, and 1 mg mL^−1^) and incubated for 10 min. The residual GSH content was then determined using a commercial assay kit, which was purchased from Biosharp Co., Ltd. (Beijing, China).

#### 2.7.4. Assessment of Ag^+^ Ions Release

A freshly prepared Ag-MOF suspension (1 mg mL^−1^) was incubated at 37 °C for 10 h, followed by centrifugation. The Ag^+^ ions concentration in the supernatant was quantified using inductively coupled plasma optical emission spectrometry (ICP-OES).

#### 2.7.5. Quantification of Nucleic Acid and Protein Leakage

*S. enteritidis* suspension was adjusted to an optical density of 0.5 at a wavelength of 600 nm (OD_600_). Then, 200 μL of Ag-MOF suspension (1 mg mL^−1^) was mixed with 200 μL of bacterial suspension and incubated at 37 °C for 120 min. Supernatants (200 μL) were collected after centrifugation, and the concentrations of nucleic acids and proteins were quantified using an ultra-microspectrophotometer (SMA1000, Merinton, Beijing, China).

### 2.8. Eradication of S. enteritidis in Food Matrices Using Ag-MOF

Food samples, including fresh pork, milk, and eggs, were purchased from a local Yangzijin supermarket in Yangzhou, China. All samples were exposed to ultraviolet irradiation for 15 min beforehand. First, 15 g of pork, 30 mL of milk, and one egg were inoculated with *S. enteritidis* suspension (10^8^ CFU mL^−1^), and then each was sprayed with approximately 1 mL of Ag-MOF suspension (1 mg mL^−1^). The samples were stored at 37 °C for 24 h. After that, the pork samples were homogenized using a blender. An amount of 5 g of homogenate was mixed with 9 mL of sterile physiological saline. An amount of 100 μL of suspension was spread onto *Salmonella* selective agar plates and incubated at 37 °C until visible colonies appeared for enumeration. The milk samples were diluted at a 1:1 ratio with sterile physiological saline, and 100 μL of suspension was plated onto *Salmonella* selective agar and incubated at 37 °C for colony counting. The egg samples were washed with 50 mL of sterile physiological saline, and 100 μL of wash solution was spread onto *Salmonella* selective agar plates and incubated at 37 °C until colony formation. Then, the eradication rate was calculated using the equation Eradication rate (%) = (*N*_control_ − *N*_treated_)/*N*_control_ × 100%, where *N*_control_ and *N*_treated_ represent the CFU in untreated and Ag-MOF-treated samples, respectively.

## 3. Results and Discussion

### 3.1. Synthesis and Characterization of Ag-MOF

**Figure 1:**
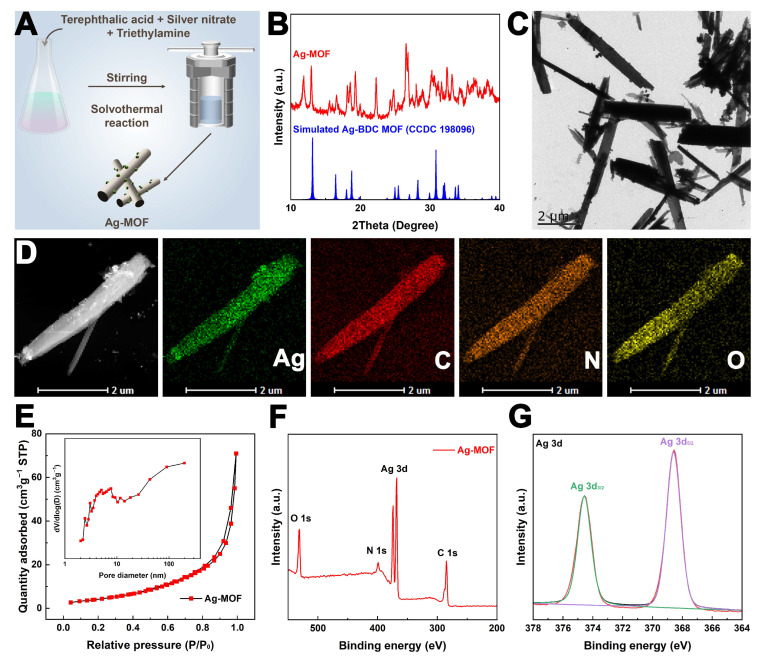
Preparation process (**A**), XRD spectrum (**B**), TEM image (**C)**, TEM element mapping images (**D**), N_2_ adsorption and desorption isotherms and pore distribution (**E**), full XPS spectrum (**F**), and high-definition Ag 3d XPS spectrum (**G**) of Ag-MOF.

Ag-MOF was synthesized via a solvothermal method with slight modifications based on a previously reported procedure (Figure 1A) [30]. The obtained Ag-MOF was first characterized using XRD. As shown in Figure 1B, the diffraction peaks of Ag-MOF are consistent with those of Ag-BDC MOF (CCDC 198096) [33], confirming its successful formation. After that, TEM images reveal that Ag-MOF exhibits rod-like nanostructures with an average length of approximately 5 µm (Figure 1C). Furthermore, elemental mapping analysis demonstrates the uniform distribution of Ag, C, N, and O elements throughout the nanorods, further validating the successful synthesis of Ag-MOF (Figure 1D). The porosity of Ag-MOF was investigated by N_2_ adsorption and desorption experiments. As shown in Figure 1E, the isotherm corresponds to a type IV curve with an H3 hysteresis loop, indicative of a mesoporous structure. Based on the Brunner–Emmet–Teller (BET) model, the specific surface area (S_BET_) was calculated to be 32.42 m^2^ g^−1^. The pore size distribution confirms the presence of abundant mesopores with a peak around 8 nm, which is favorable for its enzyme-like catalytic activity (Figure 1E inset). In addition, the surface composition of Ag-MOF was analyzed by XPS. The presence of Ag, C, N, and O elements is consistent with the TEM elemental mapping results, providing further confirmation of successful Ag-MOF synthesis (Figure 1F). As shown in Figure 1G, the Ag 3d XPS spectrum shows characteristic peaks at approximately 368.6 eV (Ag 3d_5/2_) and 374.5 eV (Ag 3d_3/2_) [34]. The binding energies suggest the possible presence of silver in different chemical states within the Ag-MOF framework, although precise quantification of Ag^0^ and Ag^+^ species was not performed. Such a heterogeneous silver chemical environment may be relevant to the redox-related catalytic behavior of Ag-MOF and is consistent with its observed oxidase-, peroxidase-, and superoxide dismutase-like activities. In addition, the presence of coordinated silver species provides a plausible basis for the gradual release of Ag^+^ ions, which may contribute to the antibacterial activity of the material.

### 3.2. Multiple Enzyme-like Activities of Ag-MOF

**Figure 2:**
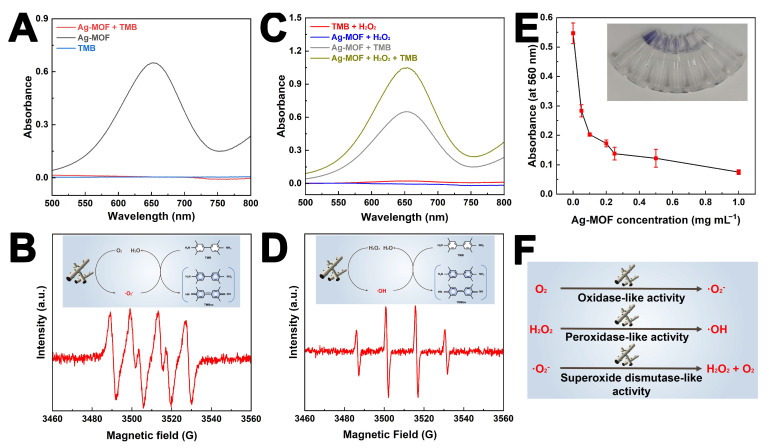
Absorbance spectra of Ag-MOF + TMB, Ag-MOF, and TMB reaction systems (**A**); EPR spectrum of DMPO + Ag-MOF (inset represents the oxidase-like mechanism of Ag-MOF) (**B**); absorbance spectra of TMB + H_2_O_2_, Ag-MOF + H_2_O_2_, Ag-MOF +TMB, and Ag-MOF + H_2_O_2_ + TMB reaction systems (**C**); EPR spectrum of DMPO + Ag-MOF + H_2_O_2_ (inset represents the peroxidase-like mechanism of Ag-MOF) (**D**); evaluation of superoxide anion scavenging capacity of Ag-MOF in the xanthine–xanthine oxidase system (**E**); and multiple enzyme-like activities of Ag-MOF (**F**).

The multiple enzyme-like activities of Ag-MOF provide a solid foundation for the effective eradication of drug-resistant *S. enteritidis*. First, the oxidase-like activity of Ag-MOF was evaluated. As shown in Figure 2A, the Ag-MOF + TMB reaction system exhibited a strong absorption peak at 652 nm, corresponding to the formation of oxidized TMB (TMBox, blue product). In contrast, no obvious absorption peak at 652 nm was observed in the TMB or Ag-MOF reaction systems alone. This result indicates that Ag-MOF can accelerate the TMB chromogenic reaction. Furthermore, the generated free radicals were detected by EPR. As shown in Figure 2B, the EPR spectrum displayed peaks consistent with the characteristic signals of superoxide anions, confirming that Ag-MOF catalyzes the generation of superoxide anions from dissolved oxygen, thereby promoting the TMB chromogenic reaction (Figure 2B inset) [35]. In addition, as shown in Figure 2C, the Ag-MOF + H_2_O_2_ + TMB reaction system displayed a much stronger absorption peak at 652 nm compared to the Ag-MOF + TMB system, suggesting that the presence of H_2_O_2_ further enhances the catalytic activity of Ag-MOF. EPR analysis further revealed the formation of hydroxyl radicals in the DMPO + Ag-MOF + H_2_O_2_ system (Figure 2D inset) [36]. These results demonstrate that Ag-MOF exhibits peroxidase-like activity, catalyzing the decomposition of H_2_O_2_ to generate hydroxyl radicals, which in turn accelerate the TMB chromogenic reaction. Moreover, the superoxide anion scavenging capacity of Ag-MOF was examined using the xanthine–xanthine oxidase system. As illustrated in Figure 2E, the absorbance at 580 nm decreased progressively with increasing concentrations of Ag-MOF, indicating its superoxide dismutase-like activity in eliminating superoxide anions [32]. Notably, the superoxide dismutase (SOD)-like activity of Ag-MOF does not eliminate oxidative stress but instead modulates ROS transformation. By catalyzing the conversion of superoxide anions into H_2_O_2_, which can subsequently generate more toxic hydroxyl radicals under peroxidase-like catalytic conditions, Ag-MOF ultimately amplifies intracellular oxidative damage rather than mitigating it.

From a mechanistic perspective, the coexistence of OXD-, POD-, and SOD-like activities endows Ag-MOF with a unique ROS-regulation capability rather than a single ROS-generation pathway. Unlike conventional antibacterial agents that rely on one dominant oxidative species, the Ag-MOF nanozyme establishes a dynamic redox cascade, enabling continuous interconversion among different ROS types under physiological conditions. Specifically, as shown in Figure 2F, oxidase-like activity facilitates the direct activation of dissolved oxygen to generate superoxide anions, while superoxide dismutase-like activity catalyzes their conversion into hydrogen peroxide. Subsequently, the peroxidase-like activity of Ag-MOF promotes the decomposition of hydrogen peroxide into highly reactive hydroxyl radicals. This sequential ROS transformation ensures sustained oxidative pressure on bacterial cells, even under fluctuating microenvironmental conditions. Such a multi-enzymatic ROS cascade is particularly advantageous in antibacterial applications as it minimizes the likelihood of bacterial adaptation to a single oxidative stress mechanism. In summary, Ag-MOF displays multiple enzyme-like activities (including OXD-, POD-, and SOD-like functions), which collectively contribute to its potential in combating drug-resistant *S. enteritidis*.

### 3.3. Eradication Activity of Drug-Resistant S. enteritidis Using Ag-MOF

**Figure 3:**
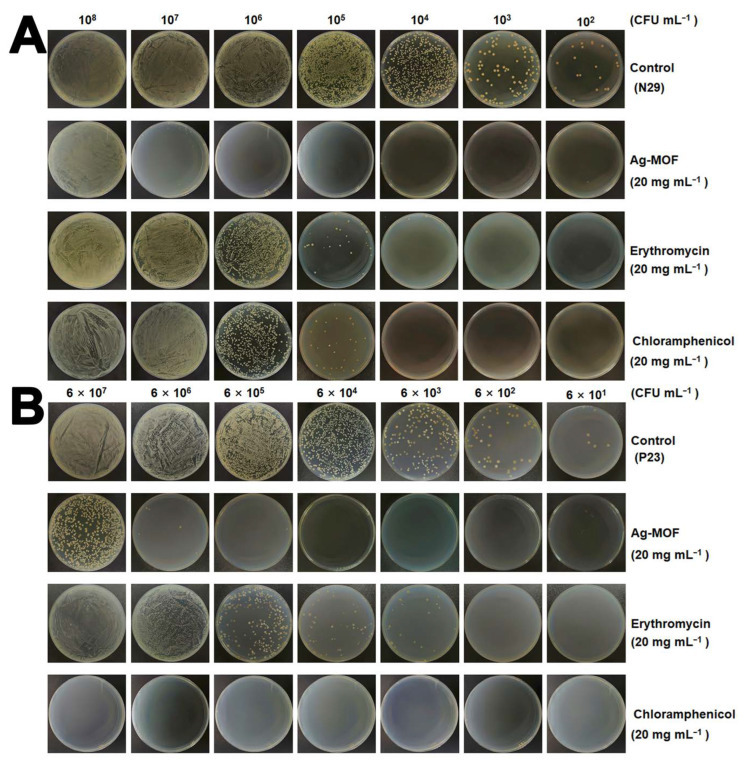
Real images of *S. enteritidis* N29 (**A**) and P23 (**B**) strains treated with Ag-MOF, erythromycin, and chloramphenicol.

Owing to its multiple enzyme-like activities, Ag-MOF was employed to combat drug-resistant *S. enteritidis*. Strains N29 and P23 were selected as representative models. Specifically, strain N29 is resistant to erythromycin and chloramphenicol, whereas strain P23 exhibits resistance only to erythromycin. Notably, erythromycin and chloramphenicol are commonly used in both clinical and agricultural settings. As shown in Figure 3A, treatment with 20 mg mL^−1^ Ag-MOF resulted in the almost complete eradication of *S. enteritidis* N29, even at high bacterial concentrations (1 × 10^7^ CFU mL^−1^). In contrast, erythromycin and chloramphenicol exhibited negligible antibacterial activity against this strain. Similarly, as illustrated in Figure 3B, Ag-MOF at the same concentration effectively eliminated *S. enteritidis* P23 (6 × 10^6^ CFU mL^−1^), whereas chloramphenicol showed strong bactericidal efficacy and erythromycin remained largely ineffective. These results indicate that Ag-MOF has excellent antibacterial activity against drug-resistant *S. enteritidis* strains.

**Figure 4:**
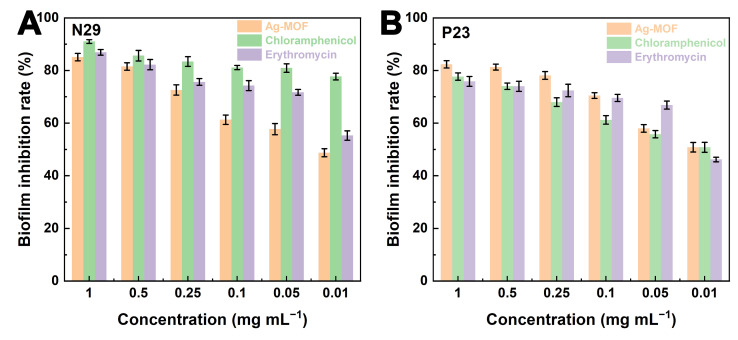
Inhibition rates of biofilm formation of *S. enteritidis* N29 (**A**) and P23 (**B**) strains using Ag-MOF, erythromycin, and chloramphenicol.

Then, the crystal violet assay was employed to further assess the inhibitory effects of Ag-MOF, erythromycin, and chloramphenicol on biofilm formation. As shown in Figure 4A,B, Ag-MOF exhibited significant biofilm inhibition in both N29 and P23 strains, with inhibition rates approaching 80~90% at 1 mg mL^−1^ and remaining 50% ~ 60% even at 0.01 mg mL^−1^. The inhibitory performance of Ag-MOF is comparable to that of erythromycin and chloramphenicol.

It is noteworthy that the outstanding antibacterial performance of Ag-MOF against drug-resistant *S. enteritidis* is distinct from that of conventional antibiotics. Erythromycin and chloramphenicol exert their bacteriostatic or bactericidal effects by targeting specific intracellular biomolecules, such as ribosomal subunits [37]. In contrast, Ag-MOF induces non-specific but highly destructive oxidative damage at the cellular level. This difference is of particular significance for combating MDR strains because the loss or modification of antibiotic targets does not undermine the antibacterial efficacy of Ag-MOF. Moreover, the robust antibiofilm activity of Ag-MOF indicates its capacity to interfere with early bacterial adhesion and extracellular polymeric substance (EPS) formation, which are crucial factors contributing to antibiotic tolerance and chronic contamination in food-processing environments [38]. Collectively, these results emphasize the advantages of nanozyme-based antibacterial strategies in addressing both planktonic bacteria and biofilm-associated infections, thus offering a more comprehensive solution for controlling resistant foodborne pathogens. In conclusion, Ag-MOF exhibits potent bactericidal activity against highly MDR *S. enteritidis* and significantly inhibits biofilm formation, highlighting its great potential as an alternative antibacterial agent.

### 3.4. Eradication Mechanism of Ag-MOF Towards S. enteritidis

**Figure 5:**
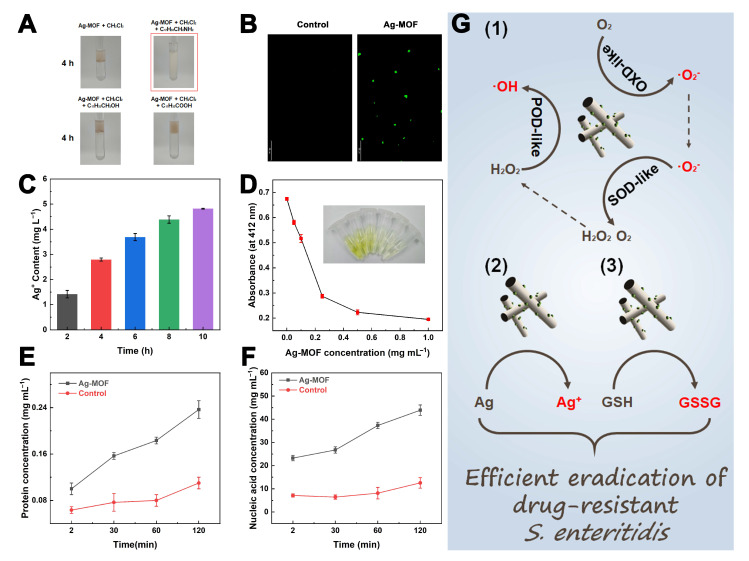
Affinity of Ag-MOF toward different functional groups (amino, hydroxyl, and carboxyl) (**A**), LSCM images showing ROS generation in *S. enteritidis* treated with Ag-MOF (**B**), Ag^+^ ions release from Ag-MOF over time (**C**), changes in absorbance of reduced glutathione after Ag-MOF treatment and corresponding real image (**D**), protein (**E**) and nucleic acid (**F**) leakage from *S. enteritidis* after Ag-MOF treatment, and antibacterial mechanism of Ag-MOF against *S. enteritidis *(**G**).

To further elucidate the antibacterial mechanism of Ag-MOF against *S. enteritidis*, a comprehensive series of experiments was conducted. First, the interactions between Ag-MOF and different functional groups (amino, hydroxyl, and carboxyl groups) were investigated. As shown in Figure 5A, Ag-MOF exhibited good dispersibility in oleyl amine but not in oleyl alcohol or oleic acid, indicating a strong affinity towards amino groups. This finding suggests that Ag-MOF may preferentially interact with amino-rich components on the bacterial surface. The Ag-MOF-induced oxidative stress was subsequently evaluated using laser scanning confocal microscopy (LSCM). As displayed in Figure 5B, intense green fluorescence was observed in *S. enteritidis* treated with Ag-MOF, confirming substantial ROS generation. These ROS are likely to be the ·O_2_^−^ and ·OH generated through Ag-MOF nanozyme. In addition, the release behavior of Ag^+^ ions from Ag-MOF was quantified, revealing a time-dependent increase (Figure 5C). The depletion of reduced glutathione (GSH) in cell-free solution by Ag-MOF was further examined. As shown in Figure 5D, the characteristic absorbance of GSH decreased with increasing Ag-MOF concentration, indicating effective extracellular oxidation of GSH to GSSG and potential suppression of the bacterial antioxidant defense system. Moreover, the leakage of intracellular components was analyzed. As depicted in Figure 5E,F, the concentrations of extracellular proteins and nucleic acids markedly increased after Ag-MOF treatment, suggesting severe damage to bacterial membranes.

In addition to oxidative stress, the physicochemical interaction between Ag-MOF and bacterial surfaces plays a crucial role in initiating antibacterial activity. The preferential affinity of Ag-MOF toward amino groups suggests strong interactions with surface proteins and peptidoglycan components, which may promote close contact between the nanozyme and bacterial membranes. This localized accumulation of Ag-MOF facilitates site-specific ROS generation and Ag^+^ ion release at the cell envelope, thereby amplifying membrane damage. Meanwhile, the depletion of intracellular GSH further exacerbates oxidative stress by impairing the bacterial redox buffering system. As GSH is a key antioxidant molecule involved in detoxifying ROS, its consumption shifts the intracellular redox balance toward a highly oxidative state, making bacterial cells more vulnerable to ROS-induced damage. Notably, the synergistic interplay among ROS generation, Ag^+^ release, and antioxidant depletion forms a self-reinforcing antibacterial loop in which membrane disruption accelerates Ag^+^ influx and ROS penetration, ultimately leading to irreversible cellular collapse.

In summary, Ag-MOF exhibits a multifaceted antibacterial mechanism (Figure 5G): (1) generation of abundant ROS via its oxidase (OXD)-, peroxidase (POD)-, and superoxide dismutase (SOD)-like catalytic activities; (2) continuous release of bactericidal Ag^+^ ions; and (3) depletion of intracellular GSH, leading to oxidative imbalance and membrane damage. These synergistic effects collectively disrupt bacterial membranes and ultimately result in the efficient eradication of *S. enteritidis*. Mechanistic studies were conducted at lower concentrations to elucidate the antibacterial pathways, which are expected to be amplified at higher dosages used for eradication.

### 3.5. Eradication of S. enteritidis in Food Matrices Using Ag-MOF

**Figure 6:**
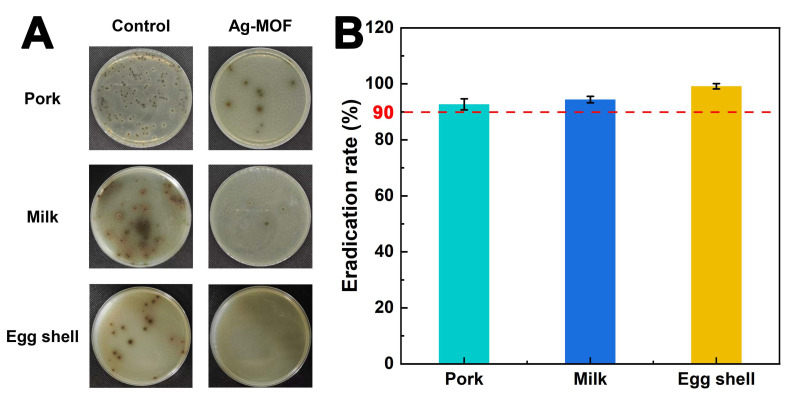
Real images of eradication of *S. enteritidis* using Ag-MOF in food matrices (**A**) and the corresponding eradication rate of Ag-MOF towards *S. enteritidis *(**B**).

The practical application of Ag-MOF in food matrices was performed. As illustrated in Figure 6A, Ag-MOF exhibited remarkable antibacterial activity against *S. enteritidis* across various food matrices, including pork, milk, and egg shell. In the control groups, numerous visible colonies were observed on the selective medium, indicating extensive proliferation of *S. enteritidis*. In contrast, almost no colonies were detected in the Ag-MOF-treated groups, demonstrating its highly efficient bactericidal performance. Quantitative analysis presented in Figure 6B further corroborates these findings, revealing that Ag-MOF achieved eradication rates exceeding 90% in all tested samples. This exceptional antibacterial efficacy is attributed to the synergistic effects of Ag-MOF, including generation of reactive oxygen species (ROS), continuous release of Ag^+^ ions, and depletion of intracellular glutathione (GSH). The robust antibacterial efficacy of Ag-MOF in diverse food matrices further highlights its practical relevance. Unlike simplified laboratory media, real food systems contain complex organic components, such as proteins, fats, and carbohydrates, which often interfere with antibacterial agents by scavenging reactive species or blocking active sites. The retained activity of Ag-MOF under these conditions indicates its strong resistance to matrix-induced deactivation. Moreover, the spray-based application strategy employed in this study closely mimics realistic food decontamination processes, such as surface sanitation during food processing and storage. This practical compatibility, combined with the high eradication efficiency, suggests that Ag-MOF has considerable potential for integration into existing food safety management systems. Collectively, these results underscore the significant potential of Ag-MOF as a versatile antibacterial agent for enhancing food safety in diverse real-world applications.

Nevertheless, several limitations of Ag-MOF should be acknowledged. As a noble-metal-based material, the relatively high cost of silver may limit large-scale applications, although the silver content in Ag-MOF is low. The biosafety of silver-based nanomaterials, particularly potential safety concerns associated with Ag^+^ release, requires further systematic evaluation before they can be used in food-related applications. In addition, the complex components of food matrices may influence the stability and antibacterial efficiency of Ag-MOF, and issues related to reusability and environmental fate also require further investigation. In addition, the application of Ag-MOF may potentially alter certain intrinsic properties of foods, such as surface appearance, texture, or physicochemical stability, which constitutes an additional limitation that should be carefully evaluated in future studies.

## 4. Conclusions

In summary, a multiple enzyme-like Ag-MOF nanozyme with oxidase-, peroxidase-, and superoxide dismutase-like activities was successfully developed for the efficient eradication of drug-resistant *S. enteritidis*. The Ag-MOF exhibited remarkable bactericidal and biofilm inhibition abilities, driven by the synergistic generation of reactive oxygen species (ROS), continuous release of Ag^+^ ions, and depletion of intracellular glutathione (GSH). These combined mechanisms disrupted bacterial membranes and impaired cellular integrity, leading to the complete inactivation of resistant *S. enteritidis*. Furthermore, Ag-MOF demonstrated strong potential for practical applications in real food matrices, offering a promising strategy to control foodborne pathogens and enhance food safety.

## Data Availability

The data presented in this study are available upon request from the corresponding authors.

## References

[1] Li Y., Qiao J., Zhao Z., Zhang Q., Zhang W., Man S., Ye S., Chen K., Ma L. (2023). A CRISPR/dCas9-enabled, on-site, visual, and bimodal biosensing strategy for ultrasensitive and self-validating detection of foodborne pathogenic bacteria. Food Front..

[2] Li S., He Y., Mann D.A., Deng X. (2021). Global spread of *Salmonella enteritidis* via centralized sourcing and international trade of poultry breeding stocks. Nat. Commun..

[3] Ward L.R., Threlfall J., Smith H.R., O’Brien S.J. (2000). *Salmonella enteritidis* Epidemic. Science.

[4] Qin X., Lu Y., Luo Y., Cui Y., Zhao K., He Y., ur Rahman M.A., Min S., Wang W., Yang F. (2025). Alfalfa Flavonoids Mitigate *Salmonella*-Induced Colitis via the Keap1-Nrf2 and TLR4/NF-κB/COX-2 Pathways. Food Front..

[5] Howard Z.R., O’Bryan C.A., Crandall P.G., Ricke S.C. (2012). *Salmonella enteritidis* in shell eggs: Current issues and prospects for control. Food Res. Int..

[6] Cai L.-L., Xie Y.-T., Hu H.-J., Xu X.-L., Wang H.-H., Zhou G.-H. (2024). Multi-omics approaches reveal inflammatory response and intestinal damage mediated by sRNA SaaS during *Salmonella* invasion in mice. Food Front..

[7] Threlfall E.J., Wain J., Peters T., Lane C., De Pinna E., Little C.L., Wales A.D., Davies R.H. (2014). Egg-borne infections of humans with salmonella: Not only an *S. enteritidis* problem. World’s Poult. Sci. J..

[8] Walker J., Chaguza C., Grubaugh N.D., Carey M., Baker S., Khan K., Bogoch I.I., Pitzer V.E. (2023). Assessing the global risk of typhoid outbreaks caused by extensively drug resistant *Salmonella typhi*. Nat. Commun..

[9] Bisola Bello A., Olamilekan Adesola R., Idris I., Yawson Scott G., Alfa S., Akinfemi Ajibade F. (2024). Combatting extensively drug-resistant *Salmonella*: A global perspective on outbreaks, impacts, and control strategies. Pathog. Glob. Health.

[10] Parmanik A., Das S., Kar B., Bose A., Dwivedi G.R., Pandey M.M. (2022). Current Treatment Strategies Against Multidrug-Resistant Bacteria: A Review. Curr. Microbiol..

[11] Soni A., Brightwell G. (2024). Genetic determinants of thermal resistance in foodborne bacterial pathogens. Food Saf. Health.

[12] Makabenta J.M.V., Nabawy A., Li C.-H., Schmidt-Malan S., Patel R., Rotello V.M. (2021). Nanomaterial-based therapeutics for antibiotic-resistant bacterial infections. Nat. Rev. Microbiol..

[13] Kurt Yilmaz N., Schiffer C.A. (2021). Introduction: Drug Resistance. Chem. Rev..

[14] Maillard J.-Y., Pascoe M. (2024). Disinfectants and antiseptics: Mechanisms of action and resistance. Nat. Rev. Microbiol..

[15] Li J., Zhao F., Zhan W., Li Z., Zou L., Zhao Q. (2022). Challenges for the application of bacteriophages as effective antibacterial agents in the food industry. J. Sci. Food Agric..

[16] Jin L., Cao F., Gao Y., Zhang C., Qian Z., Zhang J., Mao Z. (2023). Microenvironment-Activated Nanozyme-Armed Bacteriophages Efficiently Combat Bacterial Infection. Adv. Mater..

[17] Diao Q., Chen X., Tang Z., Li S., Tian Q., Bu Z., Liu H., Liu J., Niu X. (2024). Nanozymes: Powerful catalytic materials for environmental pollutant detection and degradation. Environ. Sci. Nano.

[18] Gao L., Zhuang J., Nie L., Zhang J., Zhang Y., Gu N., Wang T., Feng J., Yang D., Perrett S. (2007). Intrinsic peroxidase-like activity of ferromagnetic nanoparticles. Nat. Nanotechnol..

[19] Zhang Z., Tang L., Sheng W., Yang Y., Lai G., Liu J., Niu X. (2025). A nanozyme catalysis-diazotization reaction cascaded ratiometric colorimetric assay probing the dynamics of nitrite in leftovers. Food Saf. Health.

[20] Xu X., Luo Z., Ye K., Zou X., Niu X., Pan J. (2021). One-pot construction of acid phosphatase and hemin loaded multifunctional metal–organic framework nanosheets for ratiometric fluorescent arsenate sensing. J. Hazard. Mater..

[21] Xu X., Luo J., Wei S., Zou X., Niu X., Pan J. (2020). Three-dimensional flower-like multifunctional adsorbents with excellent sorptive removal and colorimetric detection of arsenate. Chem. Eng. J..

[22] Wu J., Wang X., Wang Q., Lou Z., Li S., Zhu Y., Qin L., Wei H. (2019). Nanomaterials with enzyme-like characteristics (nanozymes): Next-generation artificial enzymes (II). Chem. Soc. Rev..

[23] Xu X., Yang J., Hao G., Wang B., Ma T., Zhu S., Gao L., Yang Z.-Q. (2025). Three in one: A multifunctional oxidase-mimicking Ag/Mn_3_O_4_ nanozyme for colorimetric determination, precise identification, and broad-spectrum inactivation of foodborne pathogenic bacteria. Food Chem..

[24] Yu L., Sun Y., Niu Y., Zhang P., Hu J., Chen Z., Zhang G., Xu Y. (2023). Microenvironment-Adaptive Nanozyme for Accelerating Drug-Resistant Bacteria-Infected Wound Healing. Adv. Healthc. Mater..

[25] Xu Y., Luo Y., Weng Z., Xu H., Zhang W., Li Q., Liu H., Liu L., Wang Y., Liu X. (2023). Microenvironment-Responsive Metal-Phenolic Nanozyme Release Platform with Antibacterial, ROS Scavenging, and Osteogenesis for Periodontitis. ACS Nano.

[26] Zhou B., Wang Z., Sun X., Sun J., Hu S., Sun L., Xu L., Bai X., Liu M., Wang L. (2025). Nanozyme platform with built-in electric field and gas modulation for deep biofilm penetration and enhanced antibacterial therapy. Chem. Eng. J..

[27] Yao H., Zhou R., Wang J., Wei Y., Li S., Zhang Z., Du X.-D., Wu S., Shi J. (2023). Pathogen-Targeting Bimetallic Nanozymes as Ultrasonic-Augmented ROS Generator against Multidrug Resistant Bacterial Infection. Adv. Healthc. Mater..

[28] Hassan P.B., Mohammed Ameen S.S., Mohammed L., Muhammed Ameen S.M., Omer K.M. (2024). Enhanced antibacterial activity of a novel silver-based metal organic framework towards multidrug-resistant *Klebsiella pneumonia*. Nanoscale Adv..

[29] Gao L., Zhang L., Yang J., Ma T., Wang B., Yang H., Lin F., Xu X., Yang Z.-q. (2024). Immobilization of a broad host range phage on the peroxidase-like Fe-MOF for colorimetric determination of multiple *Salmonella enterica* strains in food. Microchim. Acta.

[30] Mohammed Ameen S.S., Omer K.M. (2024). Pushing Boundaries: Introducing Silver-Based Metal–Organic Framework Oxidase-Like Nanozyme over a Wide-Range Temperature. ACS Appl. Nano Mater..

[31] Tan M., Li X., Gao L., Hu Q., Yang Z., Xu X. (2025). Simultaneous detection of multispecies foodborne pathogens using multiple phage-functionalized nanozyme. J. Hazard. Mater..

[32] Xu X., Ma T., Gao Q., Tan M., Cen B., Hu Q., Gao L., Yang Z.-Q. (2025). A multiple enzyme-mimicking Ag/Fe-ZIF nanozyme for efficient inactivation and colorimetric sensing of foodborne pathogens. Chem. Eng. J..

[33] Wang T., Chen R., Li Y., Guo C., Li C. (2023). AgZn-BDC-derived catalyst for CO_2_ electroreduction to syngas with fixed ratio over wide current density. Chem. Eng. Sci..

[34] Wei F., Cui X., Wang Z., Dong C., Li J., Han X. (2021). Recoverable peroxidase-like Fe_3_O_4_@MoS_2_-Ag nanozyme with enhanced antibacterial ability. Chem. Eng. J..

[35] Tan S., Tan M., Zhang T., Gao L., Li Y., Xu X., Yang Z. (2024). Establishment of a visual assessment platform for total antioxidant capacity of *Ganoderma sichuanense* based on Arg-CeO_2_ hydrogel nanozyme utilizing a self-programmed smartphone app and a self-designed 3D-printed device. Microchim. Acta.

[36] Gao L., Long Q., Cen B., Gao Q., Tan M., Zhang L., Yang J., Ma Y., Xu X., Yang Z. (2025). Immobilization of a novel bacteriophage PpZDSS02 onto the peroxidase-mimicking Cu-MOF for colorimetric sensing of *Proteus penneri* encompassing both promotion and inhibition mechanisms. Food Chem..

[37] Deng H., McShan D., Zhang Y., Sinha S.S., Arslan Z., Ray P.C., Yu H. (2016). Mechanistic Study of the Synergistic Antibacterial Activity of Combined Silver Nanoparticles and Common Antibiotics. Environ. Sci. Technol..

[38] Wang Y., Yang Y., Shi Y., Song H., Yu C. (2020). Antibiotic-Free Antibacterial Strategies Enabled by Nanomaterials: Progress and Perspectives. Adv. Mater..

